# Genomic Analysis to Elucidate the Lignocellulose Degrading Capability of a New Halophile *Robertkochia solimangrovi*

**DOI:** 10.3390/genes13112135

**Published:** 2022-11-17

**Authors:** Ming Quan Lam, Nicola C. Oates, Daniel R. Leadbeater, Kian Mau Goh, Adibah Yahya, Madihah Md Salleh, Zaharah Ibrahim, Neil C. Bruce, Chun Shiong Chong

**Affiliations:** 1 Department of Biosciences, Faculty of Science, Universiti Teknologi Malaysia, Skudai 81310, Johor, Malaysia; 2 Centre for Novel Agricultural Products, Department of Biology, University of York, York YO10 5DD, UK

**Keywords:** *Robertkochia solimangrovi*, lignocellulolytic enzymes, genomics, halophiles, mangrove microbiota

## Abstract

*Robertkochia solimangrovi* is a proposed marine bacterium isolated from mangrove soil. So far, the study of this bacterium is limited to taxonomy only. In this report, we performed a genomic analysis of *R. solimangrovi* that revealed its lignocellulose degrading ability. Genome mining of *R. solimangrovi* revealed a total of 87 lignocellulose degrading enzymes. These enzymes include cellulases (GH3, GH5, GH9 and GH30), xylanases (GH5, GH10, GH43, GH51, GH67, and GH115), mannanases (GH2, GH26, GH27 and GH113) and xyloglucanases (GH2, GH5, GH16, GH29, GH31 and GH95). Most of the lignocellulolytic enzymes encoded in *R. solimangrovi* were absent in the genome of *Robertkochia marina*, the closest member from the same genus. Furthermore, current work also demonstrated the ability of *R. solimangrovi* to produce lignocellulolytic enzymes to deconstruct oil palm empty fruit bunch (EFB), a lignocellulosic waste found abundantly in palm oil industry. The metabolic pathway taken by *R. solimangrovi* to transport and process the reducing sugars after the action of lignocellulolytic enzymes on EFB was also inferred based on genomic data. Collectively, genomic analysis coupled with experimental studies elucidated *R. solimangrovi* to serve as a promising candidate in seawater based-biorefinery industry.

## 1. Introduction

Halophiles are extremophiles that require salt for growth [1]. They are equipped with adaptive mechanisms to survive in harsh osmotic conditions [2,3,4,5,6]. Halophilic microorganisms can be found in coastal and open ocean environments such as marine waters, saline lakes, and mangrove forests [7,8,9,10]. Mangrove forests contain plants that grow at the interface between land and sea [11] and are one of the world’s most extensive reservoirs of naturally sequestered carbon, accounting for 30% of blue carbon stored [12,13]. Halophytes within mangrove forests play an important role in the degradation of woody plant material present in mangrove sediments and surfaces [14,15,16,17].

Aside from an ecological role, lignocellulolytic enzymes produced by bacteria, including cellulases, hemicellulases, ligninases and pectinases are also important for pre-treatment and saccharification of lignocellulosic biomass for biorefining applications [18]. These enzymes are classified into glycosyl hydrolases (GHs), carbohydrate esterases (CEs), auxiliary activities (AAs) and polysaccharide lyases (PLs) [19]. They work together to degrade complex lignocellulosic plant materials to produce pentose and hexose sugars that can be utilized as feedstocks for the product of biofuels and chemicals [20,21,22,23]. In Malaysia and Indonesia oil palm empty fruit bunch (EFB) is a major lignocellulosic waste from the palm oil industry. Extensive efforts are being invested in using such lignocellulosic materials for bioenergy production [24,25].

*R. solimangrovi* is a halophilic bacterium recently isolated from mangrove soil [26]. So far, within the *Robertkochia* genus, *R. solimangrovi* is only the second species reported after *R. marina* [27]. To date, the understanding of this bacterium is limited. In this study, we report the genomic analysis of *R. solimangrovi* and its potential in producing lignocellulolytic enzymes for EFB degradation.

## 2. Materials and Methods

### 2.1. Whole Genome Sequencing, Assembly, and Annotation

The genome of *R. solimangrovi* was sequenced and assembled [26]. In brief, the genome of *R. solimangrovi* was sequenced using Illumina HiSeq 2500 platform (2 × 150 bp) and de novo assembled using SOAPdenovo v. 2.04. The assembled genome was then annotated and analyzed by NCBI Prokaryotic Genome Annotation Pipeline (PGAP) [28] and DOE-JGI Microbial Genome Annotation Pipeline (MGAP) [29]. The non-coding RNA genes, including rRNA, tRNA and ncRNA were predicted by using rRNAmmer [30], tRNAscan-SE [31] and Rfam [32] accordingly. The protein coding genes were predicted by GeneMarkS+ with the “best-placed reference protein set” method [33] and Prodigal [34]. Mapping of metabolic pathways was accomplished by using KEGG Automatic Annotation Server (KAAS) [35]. The whole genome sequence of *R. solimangrovi* is available at DDBJ/EMBL/GenBank and DOE-JGI Genome Online Database (GOLD) with accession number QKWN00000000 and Ga0314138, respectively.

### 2.2. Comparative Genomic Analysis

The genome of the other species belongs to the *Robertkochia* genus, *R. marina* was sequenced, assembled, and annotated similarly as *R. solimangrovi*. The genome can be accessed at DDBJ/EMBL/GenBank and DOE-JGI Genome Online Database (GOLD) with accession numbers QXMP00000000 and Ga0314139, respectively. The comparative genomic analysis of the two species was performed using Clusters of Orthologous Groups (COGs), genome alignment, homologous genes, and carbohydrate active enzymes (CAZymes). The protein coding genes encoded in both genomes were categorized according to COGs using RSP-BLAST [36] through the WebMGA server [37]. The genome organization between both *Robertkochia* spp. were compared by aligning the both genomes with the aid of Mauve v. 2.4.0 using default parameters [38]. The homologous gene clusters between both genomes were compared using OrthoVenn2, with the following settings: E-value of 0.05, inflation value of 1.5 and Markov Cluster as algorithm [39]. The *P* values for GO terms in a clusters overlapping were calculated by using hypergeometric distribution in OrthoVenn2.

### 2.3. CAZyme Screening and Analysis of Lignocellulolytic Genes

Putative CAZyme genes were annotated in the integrated dbCAN2 meta server following default settings, with dbCAN (using HMMER), CAZy (using DIAMOND) and PPR (using Hotpep) as detection methodologies [40,41]. The results obtained were downloaded and organized in R. The CAZymes were retained if they were recognized by at least two of the methods. The lignocellulolytic genes were further cross-checked with the annotations accessible in the CAZy database [19]. The lignocellulolytic genes were searched against NCBI non-redundant protein database using BLASTp to compare the similarity with the proteins available in the database. The secondary structure of the lignocellulolytic genes was predicted through GOR4 [42]. Additional features of the lignocellulolytic genes were examined using InterPro 77.0 [43].

### 2.4. Inoculum Preparation and Lignocellulolytic Enzyme Production

Oil palm empty fruit bunch (EFB) was collected from a palm oil mill located at Johor, Malaysia and was used as a lignocellulosic substrate. The EFB was washed, dried and ground into fibre form (2 mm) before use. A loopful of colonies of *R. solimangrovi* was inoculated in an Erlenmeyer flask with lignocellulolytic enzymes production medium (pH 7) containing MgCl_2_ (5.0 g/L), MgSO_4_·7H_2_O (2.0 g/L), CaCl_2_ (0.5 g/L), KCl (1.0 g/L), NaCl (20.0 g/L), yeast extract (1.0 g/L), peptone (5.0 g/L) and EFB (10.0 g/L) [41], and incubated at 30 °C with 150 rpm in an orbital shaker. When the OD_600_ >1.0, a 5% (*v*/*v*) bacterial inoculum was then transferred into a new lignocellulolytic enzymes production medium with same components and incubated under same conditions for 24 h to 96 h. Microbial growth, indicated by optical density at 600 nm (OD_600_) was measured at regular time points: every 24 h until 96 h, using a spectrophotometer (Thermo Fisher Scientific, Waltham, MA, USA). A negative control (without bacterial cells) was also prepared. The microbial growth at each time interval was calculated by: OD_600_ at each time interval minus OD_600_ of the control set. 

### 2.5. EFB Weight Loss Assessment and Lignocellulolytic Enzyme Activity Assays

The flasks with EFB and cells at each time interval (24 h, 48 h, 72 h, and 96 h) were centrifuged at 4 °C and 4500 rpm for 20 min. The remaining EFB was washed with 1× PBS buffer supplemented with 0.5 % (*v*/*v*) Tween 20. The EFB was dried at 60 °C and the weight of EFB was measured on an electronic balance until a constant weight was obtained. The weight loss as compared to control (EFB without inoculation) was calculated. The morphological changes of EFB were recorded. The structural changes of EFB were recorded, before and after the incubation, by using a Phenom Pro G5 scanning electron microscope (SEM) (Phenom-World BV) under 600× magnification and 5 kV accelerating voltage.

The supernatant obtained after centrifugation was utilized as crude enzymes for assays. The activities of nine lignocellulolytic enzymes were tested at every 24 h time interval until 96 h in the absence and presence of salt. Endoglucanase, exoglucanase, β-xylanase and β-mannanase activities were measured through the release of reducing sugars, based on a 3,5-dinitrosalicylic acid (DNS) method [44] with 1% (*w*/*v*) carboxymethyl cellulose (CMC) (Merck), Avicel^®^ (Merck), xylan from beechwood (Apollo Scientific) and locust bean gum from *Ceratonia siliqua* seeds (Sigma) as substrate, respectively. While ρ-nitrophenol (ρNP) based substrates (in 5 mM) were used to determine β-glucosidase, β-xylosidase, α-l-arabinofuranosidase, α-galactosidase and β-mannosidase activities, including ρNP-β-d-glucopyranoside (ρNPG) (Merck), pNP-β-d-xylopyranoside (ρNPX) (Merck), ρNP-β-α-l-arabinofuranoside (ρNP-Ara) (Megazyme), ρNP-α-d-galactopyranoside (ρNPGa) (Apollo Scientific) and pNP-β-d-mannopyranoside (ρNP-βM) (Apollo Scientific), correspondingly. The reaction mixtures consisted of an equal volume of crude enzymes and substrates in 50 mM sodium phosphate buffer (pH 7) and were incubated at 30 °C for 30 min. For the experiment in the presence of salt, 2% (*w*/*v*) NaCl was added into the buffer. The optical density after incubation was measured at 540 nm (for reducing sugar assays using DNS) and 430 nm (for detecting the release of ρNP) by using a spectrophotometer (Thermo Fisher Scientific). One unit of enzyme activity (U/mL) was defined as the amount of enzyme that liberates 1 µmol of the respective product under assay conditions. Enzyme relative activity (%) was calculated by relative to the case of reaction at which maximum activity was taken as 100%. The experiment was repeated twice, and each set was conducted in biological triplicates with a prepared negative control (without enzymes). 

### 2.6. Statistical Analysis

The data of biomass weight loss and enzyme activities were expressed as the mean ± SD. The student t-test was performed on the above-mentioned data by using SPSS v. 26 (SPSS Institute, Chicago, IL, USA) to examine the significant differences. A value of *p* < 0.05 was used as a criterion for statistical significance.

## 3. Results and Discussion

### 3.1. Genome Features of R. solimangrovi

The general genome features of *R. solimangrovi* are listed in Table 1. The genome of *R. solimangrovi* was 4.4 Mbp with G+C content of 40.72%. The genome of *R. solimangrovi* is exceptionally larger than its counterpart in the genus, i.e., *R. marina* (3.6 Mbp). While the G+C content of *R. solimangrovi* is slightly lower than *R. marina* (43.7%). Both species of *Robertkochia* have higher G+C percentage (40.7–43. 7%) compared to members of closely related genera such as *Joostella marina* (33.6%), *Galbibacter marinus* (37.0%) and *Zhouia amylolytica* (36.7%) [45,46,47].

Genome annotation of *R. solimangrovi* assigned 3669 protein coding genes and 51 RNA genes from a total of 3720 genes (Table 1). Notable features of the protein coding genes include the number of hypothetical proteins and putative horizontal transfer genes. A total of 1081 hypothetical proteins were encoded in the genome (29.5%), indicating a 1/3 portion of genes in *R. solimangrovi* could serve as potential candidates for new functional exploration. On the other hand, the 149 genes of annotated as horizontal transfer genes are potentially important for adaptabion of *R. solimangrovi* to its habitat. For example, a β-lactamase originated from archaeon *Methanosarcina* sp. MTP4 that has been transferred to *R. solimangrovi* could be used as a penicillin-binding protein to fight against the β-lactam antibiotics produced by other bacteria. 

### 3.2. Genome Comparison: R. solimangrovi vs. R. marina

Both genomes of *Robertkochia* spp. were compared in terms of COGs, genome alignment, homologous genes, and CAZymes. A total of 77.4% and 76.9% from *R. solimangrovi* and *R. marina* were functionally classified into 21 categories of COGs, respectively (Supplementary Materials Table S1). The composition of genes in COG functional categories appeared to be similar for both *R. solimangrovi* and *R. marina*. The genes assigned to general function prediction were the most abundant (13.4–13.6%), followed by genes classified under amino acid transport and metabolism (8.3–9.6%) and cell wall/membrane/envelope biogenesis (8.2–8.9%).

**Figure 1:**
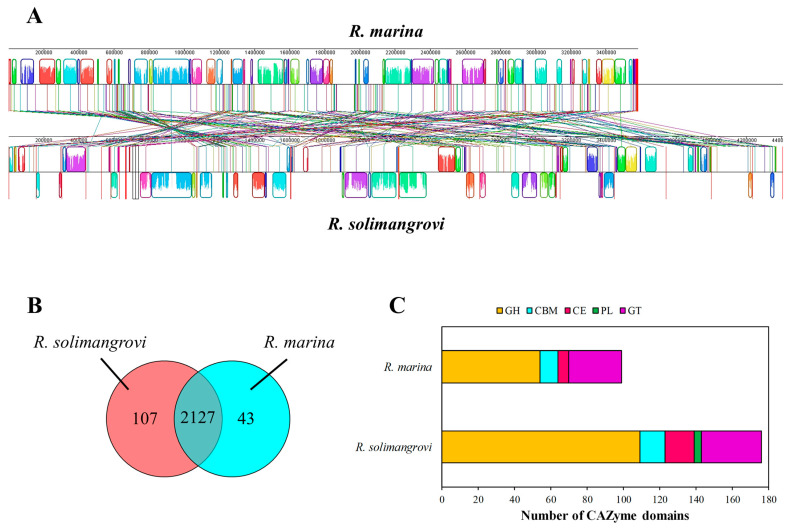
Genome alignment (**A**), clustering analysis of homologous proteins (**B**) and abundance of CAZyme domains (GHs, CEs, PLs, GTs and CBMs) encoded in genomes (**C**) of both *Robertkochia* spp.

The genome alignment of the *R. solimangrovi* and *R. marina* genomes is demonstrated in Figure 1A, with distinctive profiles for both species. The clustering analysis among both *Robertkochia* species via OrthoVenn2 indicated that they shared 2127 clusters, with another 107 paralogous clusters solely belonging to *R. solimangrovi* (Figure 1B). These paralogous gene clusters were enriched with GO-term sequence-specific DNA binding (GO:0043565), l-arabinose metabolic process (GO:0046373) and starch catabolic process (GO:00005983).

*R. solimangrovi* was comprised of 159 annotated proteins with 176 CAZyme domains, while only 93 annotated proteins with 99 CAZyme domains were found in the genome of *R. marina* (Figure 1C). Among 176 annotated CAZyme domains of *R. solimangrovi*, 109 are GHs, 14 are CBMs, 16 are CEs, 4 are PLs and 33 are glycosyl transferases (GTs). Whereas CAZyme domains of *R. marina* consisted of 54 GHs, 10 CBMs, 6 CEs and 29 GTs. Some enzymes may consist of more than one CAZyme domain [48]. A higher abundance of CAZymes was observed in the genome of *R. solimangrovi* as compared to *R. marina*, with a two-fold higher number of GHs encoded in the genome of *R. solimangrovi* and no PL was detected for *R. marina*. 

**Figure 2:**
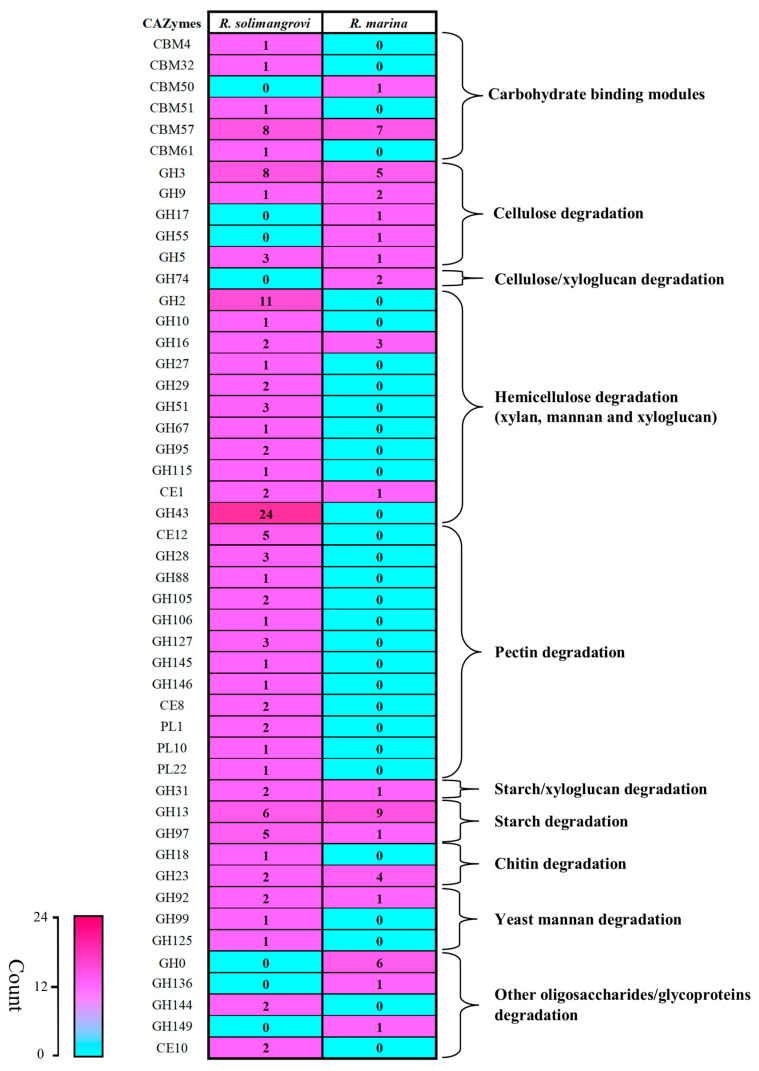
Comparative genomic analysis for polysaccharide degrading GHs, CEs, PLs and CBMs between *Robertkochia solimangrovi* and *Robertkochia marina*.

Further comparison of CAZymes among these two species elucidated a total of 48 unique differences (Figure 2). Out of the differences examined, *R. solimangrovi* possesses 30 families of CAZymes that are not present in the *R. marina*. Most of the GHs and PLs identified in the genome of *R. solimangrovi* were not present in *R. marina*. For instance, *R. solimangrovi* possesses a series of GH28, GH53, GH88, GH105, GH106, GH127, GH145, GH146, PL1, PL10 and PL22 (Figure 2) that are responsible for pectin degradation [49,50]. Interestingly, a protein that contained a PL1 with a CE8 domain was identified in *R. solimangrovi*. The combination of PL1 and CE8 in a single protein suggested that the CE8 domain could possibly de-esterify the pectate found in the mangrove environment, which then serves as the substrate for the pectin lyase PL1 domain [51]. 

### 3.3. Mining and Analysis of Lignocellulolytic Enzymes of R. solimangrovi

Further annotation of the genome of *R. solimangrovi* revealed 11 cellulases, 51 hemicellulases and 25 pectinases (Table 2). These make up a total of 87 genes that encode enzymes that are likely to be involved in lignocellulose degradation. The BLASTp search on these lignocellulose degrading genes imparted that they shared between 42.3–82.5% similarity with proteins from other genera with halophilic origin such as *Joostella*, *Flaviramulus*, *Fabibacter* and *Zhouia* (Supplementary Materials Table S2).

Among the cellulases of *R. solimangrovi* (Table 2), the GH5 sub-family 46 and GH9 are responsible for either endoglucanase/exoglucanase activities, while the GH3 and GH30 families are usually encoding for β-glucosidase activity that release glucose from cellobiose [20]. In other studies, the enzyme encoding for GH5 sub-family 46 produced by the uncultured microorganisms found in the rumen of cows, was described to be active towards carboxymethyl-cellulose [52,53].

Hemicellulases are the most abundant group of lignocellulose degrading enzymes annotated in the genome of *R. solimangrovi*, which represents the greatest difference observed between *R. solimangrovi* and *R. marina* (Figure 2). A total of 29 xylanases and 22 mannanases/xyloglucanases are encoded (Table 2). These findings indicate the potential ability of *R. solimangrovi* in hydrolyzing hemicellulose such as xylan, mannan and xyloglucan. In terms of xylan-active enzymes, 1,4-β-xylanase (GH5 sub-family 13 and GH10 with CBM22) and β-xylosidase GH43 and GH43 sub-family 31) are identified. Enzymes from these families putatively release xylose from the main xylosidic-chain [21]. Hydrolytic enzymes that cleave side chains from xylan are also present. These enzymes include acetyl xylan esterase (CE1, CE2 and CE4), α-arabinofuranosidase (GH43, GH43 sub-family 17, 18 and 19, and GH51) and α-glucuronidase (GH67 and GH115) which target the acetyl group of xylosyl unit, α-1,2/α-1,3-arabinofuranose and glucuronic acid chain linked to C_2_-OH of xylose, respectively [19]. 

A set of mannanases including β-mannanase (GH113 and GH26 + GT2 sub-family 3), β-mannosidase (GH2) and α-galactosidase (GH27) are encoded in *R. solimangrovi* (Table 2), which cooperatively to depolymerize mannan. Moreover, xyloglucanases were also present in the genome of *R. solimangrovi*, including xyloglucan-specific endo-β-1,4-glucanase (GH5 sub-family 4 and GH16), β-galactosidase (GH2), α-fucosidase (GH29 and GH95) and α-xylosidase (GH31) (Table 2). The breakdown of xyloglucan by these xyloglucanases could yield glucose and xylose for bioethanol production [54]. 

Several annotated lignocellulose degrading enzymes of *R. solimangrovi* feature additional domains that are suspected to improve enzymatic function. For instance, fibronectin type 3 domains found at the C-terminal of GH3 and GH43 sub-family 8 of *R. solimangrovi* (Supplementary Materials Figure S1), could potentially assist in cellulose hydrolysis. In addition, efficient cellulose and hemicellulose degradation also requires the action of non-catalytic CBMs to enhance the association of GHs to the substrate [55]. Several GHs encoded in the genome of strain CL23 contain CBMs, for instance, GH27 with CBM51, GH29 with CBM32 and GH43 sub-family 37 with CBM61. In addition, a GH26 of *R. solimangrovi* also possesses with a GT2 in the same gene (Supplementary Materials Figure S1). GT2 has shown to be involved in synthesis of polysaccharides such as cellulose and mannan exopolysaccharides [56]. However, the co-occurrence of both GH26 and GT2 within the same gene has not been previously reported. 

Additionally, a total of 8 lignocellulolytic enzymes including GH9, GH2, GH5 sub-family 4, GH16, GH43, GH43 sub-family 17 and 28 and GH127 were transferred from different bacteria to *R. solimangrovi* (Supplementary Materials Table S3). These horizontal transferred genes likely assist *R. solimangrovi* to metabolize lignocellulose from mangrove.

### 3.4. Capability of R. solimangrovi to Deconstruct Oil Palm Empty Fruit Bunch

The presence of lignocellulose degrading genes in *R. solimangrovi* suggests that this bacterium can deconstruct lignocellulosic materials. Therefore, *R. solimangrovi* was cultured in a medium with empty fruit bunch (EFB). Structural and weight changes of EFB were monitored. A total of 30.4% EFB biomass weight was lost after 96 h of incubation (Supplementary Materials Figure S2A), a significant amount as compared to the control (only 0.3% biomass weight loss). The EFB after degradation by *R. solimangrovi* was examined under SEM (Supplementary Materials Figure S2B). The structure of EFB was altered to broken and rough surfaces after 96 h of incubation with *R. solimangrovi* (Supplementary Materials Figure S2B(ii)) as compared to control which was smooth and intact (Supplementary Materials Figure S2B(i)).

**Figure 3:**
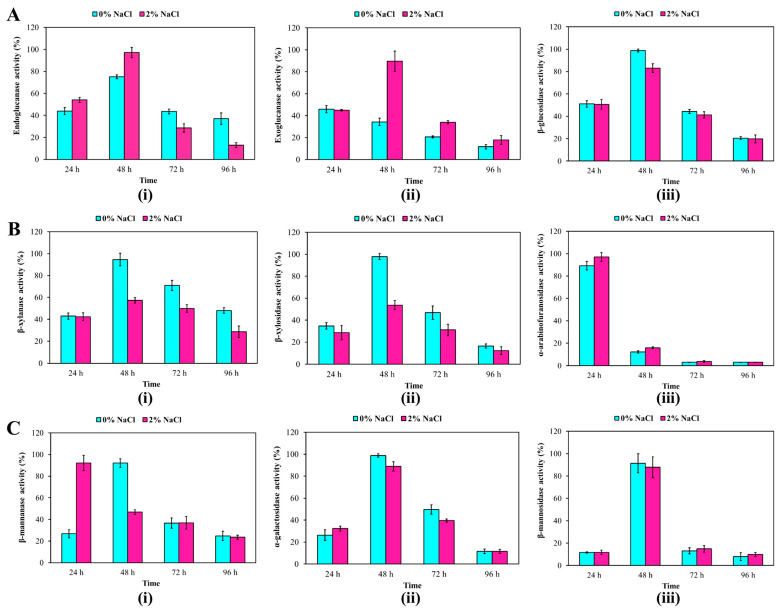
Cellulase (**A**), xylanase (**B**) and mannanase (**C**) activities of *R. solimangrovi* across 96 h of incubation with EFB. Enzyme relative activity (%) was calculated by relative to the case of reaction at which maximum activity was taken as 100%. Mean values (*n* = 3) are expressed, and standard deviations are indicated as error bars. Significant differences (*p* < 0.05) were examined by using student *t*-test.

The bacterial growth and a total of nine different lignocellulolytic enzyme activities were monitored across different incubation periods (24 h, 48 h, 72 h and 96 h) under assay conditions with or without NaCl (2% (*w*/*v*)) (Figure 3). The bacterial growth remained relatively constant from 24 h to 72 h (OD_600_ = 1.6–1.7), and decreased at 96 h (OD_600_ = 1.1). All nine lignocellulolytic enzymes activities (endoglucanase, exoglucanase, β-glucosidase, β-xylanase, β-xylosidase, α-arabinofuranosidase, β-mannanase, β-mannosidase and α-galactosidase) were produced by *R. solimangrovi* during the incubation period (24 h, 48 h, 72 h, and 96 h) (Figure 3). Based on the lignocellulolytic enzymes analysis (Table 2), GH5 sub-family 46 and GH9 family are likely to contribute to endoglucanase activity, while the GH3 and GH30 family could contribute to β-glucosidase activity. Furthermore, the xylanase activities (β-xylanase, β-xylosidase and α-arabinofuranosidase) could be potentially contributed by GH5 sub-family 13, GH10, GH51 and GH43. While GH113, GH26 and GH27 families could play a role in the activity of β-mannanase, β-mannosidase and α-galactosidase, respectively.

Highest enzyme activity was observed for α-arabinofuranosidase after 24 h of incubation. All tested lignocellulolytic enzymes demonstrated an increase of activity after 48 h of incubation (except for α-arabinofuranosidase). Reduced enzyme activities were seen for all of the tested lignocellulolytic enzymes after 72 h of incubation (Figure 3). Under assay conditions with or without NaCl (2% (*w*/*v*)), similar enzyme activities were observed for β-glucosidase, α-arabinofuranosidase, α-galactosidase and β-mannosidase across the 96 h of incubation. The β-xylanase and β-xylosidase revealed higher enzyme activities when NaCl was absent in enzyme assay buffer (at 48 h, 72 h and 96 h of incubation). The genes encoding for β-xylanase (GH5 sub-family 13 and GH10) and β-xylosidase (GH43 and GH43 sub-family 31) contain high proportion of hydrophobic amino acids. This property does not allow the enzymes to counterbalance the hydrophobic interaction strengthened by the presence of salts in the surrounding environment [57], therefore, β-xylanase and β-xylosidase showed lower tolerance to saline condition. 

In contrast, higher enzyme activities were seen for endoglucanase and exoglucanase (after 48 h of incubation) when enzyme assays were conducted in the presence of NaCl (2% (*w*/*v*). A higher acidic amino acid composition (glutamine and aspartic acid) was found in the sequences of GH5 sub-family 46 and GH9 that were likely contributed to endoglucanase and exoglucanase activities in saline conditions, respectively. In GH5, there is a total of 76 acidic amino acids as compared to 62 basic amino acids. A similar result was also observed in GH9, in which this enzyme consists of 101 acidic amino acids and 81 basic amino acids. A higher proportion of acidic acid enables the enzyme to have a stable solvation shell on its structural surfaces to prevent aggregation in presence of salt [57]. Furthermore, a significant proportion of the random coil was also predicted in GH5 sub-family 46 (45.9%) and GH9 (50.3%) according to GOR4 secondary structure prediction. This may allow the enzyme to have high degree of flexibility to prevent structural collapse under a high salt condition [57].

### 3.5. Potential Sugar Uptake and Metabolic Pathway Taken by R. solimangrovi

**Figure 4:**
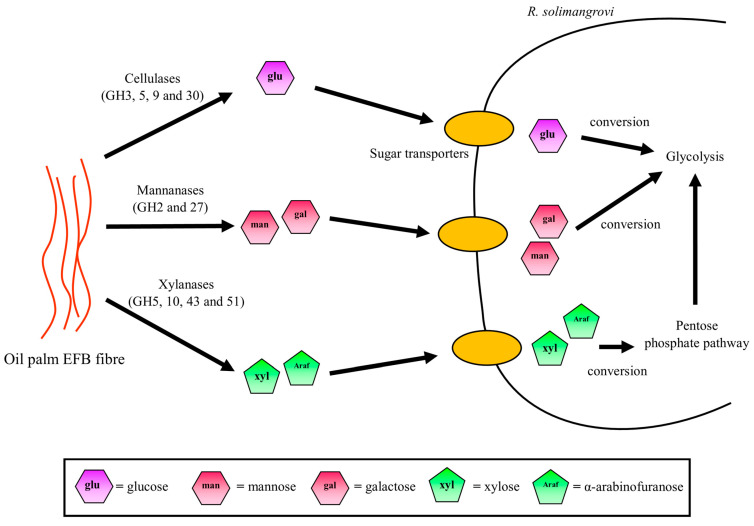
Sugar monomers transportation and metabolic pathways utilized by *R. solimangrovi*.

Upon the degradation of EFB by lignocellulolytic enzymes of *R. solimangrovi*, sugars such as glucose, mannose, galactose, xylose and arabinofuranose are produced. These could be acquired by cells as carbon sources for growth (Figure 4). Several genes that encode for transporters are found in the genome of *R. solimangrovi*. These transporters are glucose/Na^+^ co-transporters, ABC transporters, MFS transporters and EamA/RhaT family transporters (Supplementary Materials Table S4). Based on KEGG analysis, the fate of sugar monomers after transportation into the cells is different (Figure 4). The d-glucose depolymerized from the cellulose component of the EFB is in β-form [58]. In order to be utilized by cells, the β-glucose is firstly phosphorylated to β-glucose-6-phosphate by polyphosphate glucokinase and then converted to its α-form by glucose-6-phosphate isomerase (Supplementary Materials Table S4) [59]. The α-glucose-6-phosphate could then enter the glycolytic pathway for energy generation. Similarly, galactose undergoes a series of conversions into α-glucose-6-phosphate by galactose-1-epimerase, galactokinase, galactose-1-phosphate uridylyltransferase, phospho-sugar mutase (Supplementary Materials Table S4) and subsequently enters the glycolytic cycle [59].

Unlike glucose and galactose, the mannose is phosphorylated and isomerized by carbohydrate kinase and mannose-6-phosphate isomerase into β-fructose-6-phosphate (Supplementary Materials Table S4). Additional steps of conversion for pentose sugars (xylose and arabinofuranose) are required before they can enter the central metabolism. Initially, xylose is isomerized and phosphorylated into xylulose-5-phosphate by xylose isomerase and xylulokinase, respectively (Supplementary Materials Table S4). The xylulose-5-phosphate is then transformed by transketolase into β-fructose-6-phosphate in the pentose phosphate pathway and enters the glycolysis pathway. Likewise, arabinofuranose is isomerized, phosphorylated, and epimerized into xylulose-5-phosphate by arabinose isomerase, ribulokinase and ribulose-5-phosphate-4-epimerase, respectively (Supplementary Materials Table S4), and eventually glycolysis. 

Taken together, to the extent of our knowledge, this is the first genomic study of *R. solimangrovi* to reveal its lignocellulose degrading capacity. Genomic analysis coupled with experimental studies unleashed its potential to degrade lignocellulosic EFB from the oil palm industry, which could be valuable for seawater based biorefining. Future research could be directed to investigate the transcriptomic and proteomic response of *R. solimangrovi* on different lignocellulosic waste to reveal the enzymes that are highly expressed in a saline environment setting. 

## Figures and Tables

**Table 1:** General genome features of *Robertkochia solimangrovi*.

Category	Number	% of Total
Number of contigs	23	-
Genome size (bp)	4,407,290	100.00
G + C content (%)	1,794,772	40.72
Total genes predicted	3720	100.00
Protein coding genes	3669	98.63
with COGs	2840	77.41
connected to KEGG pathway	1325	36.11
RNA genes	51	1.37
rRNA genes	5	0.13
5S rRNA	1	0.03
16S rRNA	2	0.05
23S rRNA	2	0.05
tRNA	42	1.13
ncRNA	4	0.11
Pseudogenes	25	-
Horizontal transferred genes	149	4.01

**Table 2:** List of putative lignocellulose degrading enzymes encoded in genome of *R. solimangrovi*.

Category	Annotation	CAZy Family	Number of Genes	NCBI Locus Tag Accession
Cellulase	Endoglucanase/ Exoglucanase	GH5 sub-family 46	1	DMZ48_08160
		GH9	1	DMZ48_13260
	β-glucosidase	GH3	7	DMZ48_01605 DMZ48_08150 DMZ48_09610 DMZ48_13440 DMZ48_13560 DMZ48_14810 DMZ48_14815
		GH30	2	DMZ48_08155 DMZ48_08165
Xylanase	1,4-β-xylanase	GH5 sub-family 13	1	DMZ48_12810
		GH10	1	DMZ48_13420
	1,4-β-xylanase/ α-arabinofuranosidase	GH43 sub-family 29	2	DMZ48_01140 DMZ48_12865
	β-xylosidase	GH43	2	DMZ48_04615 DMZ48_15560
		GH43 sub-family 31	2	DMZ48_12790 DMZ48_12800
	β-xylosidase/ α-arabinofuranosidase	GH43 sub-family 1	1	DMZ48_13400
		GH43 sub-family 10	1	DMZ48_09800
		GH43 sub-family 26	3	DMZ48_12795 DMZ48_15550 DMZ48_18410
	α-arabinofuranosidase	GH43	1	DMZ48_15515
		GH43 sub-family 17	1	DMZ48_15280
		GH43 sub-family 18	1	DMZ48_12805
		GH43 sub-family 18 + CE10	1	DMZ48_04720
		GH43 sub-family 19	1	DMZ48_14390
		GH43 sub-family 19 + GH43	1	DMZ48_07370
		GH51	3	DMZ48_01125 DMZ48_01150 DMZ48_08775
	α-glucuronidase	GH67	1	DMZ48_13435
		GH115	1	DMZ48_04675
	Uncharacterized xylanase	GH43 sub-family 28	2	DMZ48_07195 DMZ48_17740
	Acetyl xylan esterase	CE1	1	DMZ48_16570
		CE2	1	DMZ48_07320
		CE4	1	DMZ48_03780
Mannanase	β-mannanase	GH113	1	DMZ48_11390
		GH26 + GT2 sub-family 3	1	DMZ48_06700
	α-galactosidase	GH27 with CBM51	1	DMZ48_12870
Mannanase/Xyloglucanase	β-mannosidase/ β-galactosidase	GH2	11	DMZ48_03505 DMZ48_04665 DMZ48_07180 DMZ48_07385 DMZ48_14580 DMZ48_14795 DMZ48_14865 DMZ48_15505 DMZ48_15590 DMZ48_16090 DMZ48_16435
Xyloglucanase	Xyloglucan-specific endo-β-1,4-glucanase	GH5 sub-family 4	1	DMZ48_14940
		GH16	2	DMZ48_07400 DMZ48_16450
	α-fucosidase	GH29	1	DMZ48_02920
		GH29 with CBM32	1	DMZ48_16630
		GH95	2	DMZ48_14070 DMZ48_14845
	α-xylosidase	GH31	1	DMZ48_14800
Pectinase	Endo/exo-polygalacturonase	GH28	3	DMZ48_04685 DMZ48_04700 DMZ48_09805
	Endo-α-1,5-arabinanase	GH43 sub-family 4	1	DMZ48_01145
	Endo/exo-α-1,5-arabinanase	GH43 sub-family 5	1	DMZ48_01135
		GH43 sub-family 37 with CBM61	1	DMZ48_08955
	β-1,4-endogalactanase	GH53	1	DMZ48_07175
	β-1,3-exogalactanase	GH43 sub-family 24	1	DMZ48_07375
	Unsaturated glucuronyl hydrolase	GH88	1	DMZ48_16440
	Unsaturated rhamnogalacturonyl hydrolase	GH105	2	DMZ48_02835 DMZ48_04715
	α-l-rhamnosidase	GH106	1	DMZ48_04710
	l-Rhα-α-1,4-GlcA α-rhamnohydrolase	GH145	1	DMZ48_16445
	β-arabinofuranosidase	GH127	3	DMZ48_01130 DMZ48_14480 DMZ48_15545
		GH146	1	DMZ48_12780
	Rhamnogalacturonan acetylesterase	CE12	2	DMZ48_02845 DMZ48_04660
		CE12 + CE12	1	DMZ48_04670
		CE12 + CE10	1	DMZ48_09795
	Pectin lyase	PL1 sub-family 2	1	DMZ48_09815
	Pectin/pectate lyase with esterase	PL1 sub-family 2 + CE8	1	DMZ48_09810
		PL10 + CE8	1	DMZ48_02840
	Oligogalacturonate/ oligogalacturonide lyase	PL22	1	DMZ48_04705

## Data Availability

Not applicable.

## Supplementary Materials

The following supporting information can be downloaded at: https://www.mdpi.com/article/10.3390/genes13112135/s1, Figure S1: Domain organization of GH43 sub-family 28 (A), GH3 (B) and GH26 with GT2 (C) annotated in the genome of *Robertkochia solimangrovi.* SP, signal peptide; FN3, fibronectin type 3 domain; TM, transmembrane helix; Figure S2: Oil palm empty fruit bunch (EFB) deconstruction by *R. solimangrovi* as indicated by total biomass weight loss (A) and EFB structural changes (B). Scanning electron micrographs of EFB structure before strain inoculation (i) and after 96 h of incubation (ii); Table S1: Classification of protein coding genes according to Clusters of Orthologous Groups (COGs) for *R. solimangrovi* and *R. marina;* Table S2: Similarity of lignocellulose degrading genes of *R. solimangrovi* to other related sequences based BLASTp search; Table S3: Putative horizontal transferred genes of *R. solimangrovi* related to lignocellulose degradation, inferred from genomic data; Table S4: List of potential genes involved in uptake and metabolism of sugars, inferred from genome of *R. solimangrovi*.
