# Supplementary material for: Genomic Analysis to Elucidate the Lignocellulose Degrading Capability of a New Halophile Robertkochia solimangrovi
Source: Genes (Basel). 2022 Nov 17;13(11):2135. doi: 10.3390/genes13112135 (PMC9690682; doi:10.3390/genes13112135)
Supplement: Supplementary file 1 [file genes-13-02135-s001.zip › genes-2031169-supplementary.pdf]

Supplementary materials

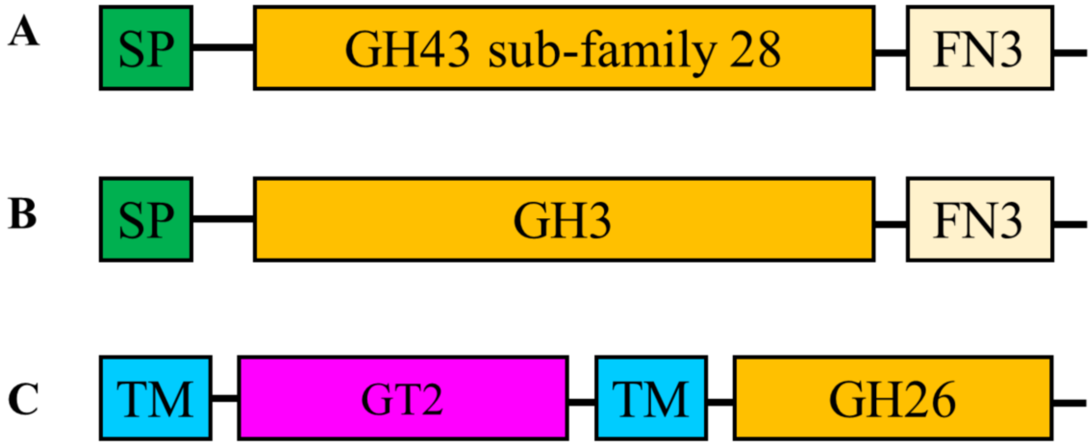

Figure S1 Domain organization of GH43 sub-family 28 (A), GH3 (B) and GH26 with GT2 (C) annotated in the genome of *Robertkochia solimangrovi*. SP, signal peptide; FN3, fibronectin type 3 domain; TM, transmembrane helix.

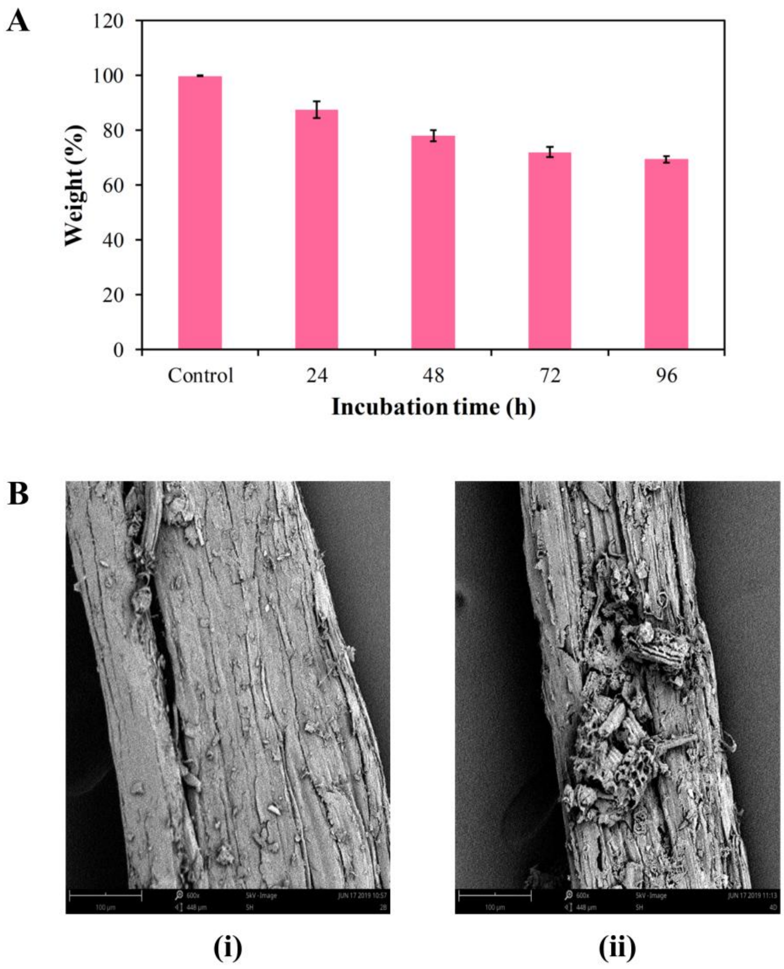

Figure S2 Oil palm empty fruit bunch (EFB) deconstruction by *Robertkochia solimangrovi* as indicated by total biomass weight loss (A) and EFB structural changes (B). Scanning electron micrographs of EFB structure before strain inoculation (i) and after 96 h of incubation (ii).

Table S1. Classification of protein coding genes according to Clusters of Orthologous Groups (COGs) for *R. solimangrovi* and *R. marina*.

Code	Description of COG functional categories	*R. solimangrovi*		*R. marina*	
		Number of genes	Percentage (%)	Number of genes	Percentag e (%)
R	General function prediction only	379	13.35	318	13.60
E	Amino acid transport and metabolism	236	8.32	225	9.59
M	Cell wall/membrane/envelope biogenesis	231	8.15	209	8.93
S	Function unknown	224	7.89	179	7.65
K	Transcription	198	6.97	119	5.09
P	Inorganic ion transport and metabolism	192	6.76	124	5.29
G	Carbohydrate transport and metabolism	186	6.55	134	5.73
C	Energy production and conversion	160	5.62	152	6.49
J	Translation, ribosomal structure and biogenesis	151	5.33	159	6.80
T	Signal transduction mechanisms	148	5.22	76	3.25
H	Coenzyme transport and metabolism	129	4.54	110	4.70
O	Posttranslational modification, protein turnover, chaperones	126	4.44	111	4.73
L	Replication, recombination and repair	101	3.54	102	4.35
I	Lipid transport and metabolism	100	3.52	89	3.79
F	Nucleotide transport and metabolism	73	2.58	69	2.95
V	Defense mechanisms	66	2.32	45	1.94
Q	Secondary metabolites biosynthesis, transport and catabolism	60	2.12	50	2.14
U	Intracellular trafficking, secretion, and vesicular transport	50	1.75	40	1.71
D	Cell cycle control, cell division, chromosome partitioning	19	0.67	21	0.91
N	Cell motility	8	0.30	7	0.32
B	Chromatin structure and dynamics	1	0.03	1	0.04

Table S2. Similarity of lignocellulose degrading genes of *R. solimangrovi* to other related sequences based BLASTp search.

CAZy family	Locus tag accession	Top hit sequence	Identity
GH5 sub-family 46	DMZ48_08160	carbohydrate binding module (family 6) of *Leeuwenhoekiella marinoflava* DSM 3653	58.00%
GH9	DMZ48_13260	glycosyl hydrolase family 9 of *Draconibacterium orientale*	63.48%
GH3	DMZ48_01605	glycosyl hydrolase of *Flavobacteriaceae* bacterium CG17_big_fil_post_rev_8_21_14_2_50_33_15	62.69%
	DMZ48_08150	beta-glucosidase of *Salinimicrobium sediminis*	77.32%
	DMZ48_09610	beta-glucosidase of *Pseudozobellia* sp. CCMM003	73.40%
	DMZ48_13440	glycosyl hydrolase of *Marivirga lumbricoides*	67.84%
	DMZ48_13560	beta-glucosidase of *Joostella marina* DSM 19592	67.63%
	DMZ48_14810	glycosyl hydrolase family 3 of *Flavobacterium subsaxonicum* WB 4.1-42	67.90%
	DMZ48_14815	glycosyl hydrolase of *Gramella* sp. SH35	60.38%
GH30	DMZ48_08155	glucosylceramidase of *Salinimicrobium sediminis*	64.03%
	DMZ48_08165	glucosylceramidase of *Flavobacteriia* bacterium	64.42%
GH5 sub-family 13	DMZ48_12810	1,4-beta-xylanase of *Salegentibacter* sp. T436	77.95%
GH10	DMZ48_13420	1,4-beta-xylanase of *Muricauda* sp. 72	63.71%
GH43 sub-family 29	DMZ48_01140	glycosyl hydrolase family 43 of *Lutibacter oceani*	75.47%
	DMZ48_12865	glycoside hydrolase of *Tamlana nanhaiensis*	84.45%
GH43	DMZ48_04615	beta-xylosidase of *Joostella marina* DSM 19592	63.94%
	DMZ48_15560	glycosyl hydrolase of *Lacibacter* sp. TTM-7	66.15%
GH43 sub-family 31	DMZ48_12790	beta-xylosidase of *Joostella marina* DSM 19592	64.78%
	DMZ48_12800	glycosyl hydrolases family 43 of *Salegentibacter salinarum*	57.06%
GH43 sub-family 1	DMZ48_13400	alpha-N-arabinofuranosidase of *Marivirga lumbricoides*	69.86%

GH43 sub-family 10	DMZ48_09800	beta-xylosidase of *Joostella marina* DSM 19592	72.31%
GH43 sub-family 26	DMZ48_12795	putative beta-xylosidase of *Joostella marina* DSM 19592	78.27%
	DMZ48_15550	glycosyl hydrolase family 43 of *Flavobacterium* sp. Leaf82	64.33%
	DMZ48_18410	alpha-N-arabinofuranosidase of *Pontibacter diazotrophicus*	68.87%
GH43	DMZ48_15515	beta-xylosidase of *Zhouia amylolytica* AD3	79.31%
GH43 sub-family 17	DMZ48_15280	glycosyl hydrolases family 43 of *Cyclobacterium lianum*	77.24%
GH43 sub-family 18	DMZ48_12805	glycosyl hydrolase family 43 of *Salegentibacter* sp. T436	80.77%
GH43 sub-family 18 + CE10	DMZ48_04720	beta-galactosidase of *Flavobacteriaceae* bacterium FS1-H7996/R	62.50%
GH43 sub-family 19	DMZ48_14390	hypothetical protein DSM04_101529 of *Leeuwenhoekiella* sp. R-50232	81.42%
GH43 sub-family 19 + GH43	DMZ48_07370	beta-xylosidase of *Joostella marina* DSM 19592	75.88%
GH51	DMZ48_01125	alpha-N-arabinofuranosidase of *Flavobacterium* sp. 123	66.92%
	DMZ48_01150	alpha-N-arabinofuranosidase of *Flavobacterium* sp. 11	76.06%
	DMZ48_08775	alpha-N-arabinofuranosidase of *Leeuwenhoekiella marinoflava* DSM 3653	60.62%
GH67	DMZ48_13435	alpha-glucuronidase of *Sinomicrobium oceani*	62.27%
GH115	DMZ48_04675	hypothetical protein IIF7_17427 of *Zunongwangia atlantica* 22II14-10F7	53.34%
GH43 sub-family 28	DMZ48_07195	F5/8 type C domain-containing protein of *Fabibacter pacificus*	81.15%
	DMZ48_17740	F5/8 type C domain-containing protein of *Porphyromonadaceae* bacterium KH3CP3RA	59.55%
CE1	DMZ48_16570	esterase family protein of *Muricauda* sp. 72	63.81%
CE2	DMZ48_07320	hypothetical protein APR42_08025 of *Salegentibacter mishustinae*	42.45%
CE4	DMZ48_03780	polysaccharide deacetylase family protein of *Tenacibaculum litoreum*	52.58%
GH113	DMZ48_11390	hypothetical protein SAMN04488008_102204 of *Maribacter orientalis*	50.49%

GH26 + GT2 sub-family 3	DMZ48_06700	family 2 glycosyl transferase of *Galbibacter marinus*	51.12%
GH27 with CBM51	DMZ48_12870	hypothetical protein PK35_02670 of *Tamlana nanhaiensis*	79.50%
GH5 sub-family 4	DMZ48_14940	aryl-phospho-beta-D-glucosidase BglC of *Cytophaga xylanolytica*	55.56%
GH2	DMZ48_03505	hypothetical protein DDZ16_13090 of *Marinilabilia* sp. WTE	61.46%
	DMZ48_04665	beta-galactosidase/beta-glucuronidase of *Joostella marina* DSM 19592	70.24%
	DMZ48_07180	beta-galactosidase of *Flaviramulus basaltis*	75.19%
	DMZ48_07385	beta-galactosidase of *Pustulibacterium marinum*	68.82%
	DMZ48_14580	beta-glycosidase of *Zunongwangia atlantica* 22II14-10F7	67.31%
	DMZ48_14795	DUF4982 domain-containing protein of *Sinomicrobium pectinilyticum*	65.00%
	DMZ48_14865	beta-galactosidase of *Gramella* sp. SH35	52.13%
	DMZ48_15505	beta-galactosidase of *Reichenbachiella* sp. 5M10	63.13%
	DMZ48_15590	beta-galactosidase of *Joostella marina* DSM 19592	75.16%
	DMZ48_16090	beta-galactosidase of *Zhouia amylolytica*	57.00%
	DMZ48_16435	beta-glucuronidase of *Mariniflexile* sp. TRM1-10	74.84%
GH43 sub-family 24	DMZ48_07375	glycosyl hydrolases family 43 of *Pustulibacterium marinum*	72.27%
GH29	DMZ48_02920	alpha-L-fucosidase of *Flavobacteriia* bacterium	62.30%
GH29 with CBM32	DMZ48_16630	alpha-L-fucosidase of *Arenibacter* sp. C-21	68.04%
GH95	DMZ48_14070	alpha-L-fucosidase of *Mangrovimonas* sp. DI 80	63.26%
	DMZ48_14845	alpha-L-fucosidase 2 of *Lutibacter flavus*	60.86%
GH31	DMZ48_14800	alpha-xylosidase of *Confluentibacter* sp. 3B	79.80%
GH16	DMZ48_07400	hypothetical protein SAMN02927921_00688 of *Sinomicrobium oceani*	57.43%
	DMZ48_16450	hypothetical protein JoomaDRAFT_0386 of *Joostella marina* DSM 19592	71.50%

GH28	DMZ48_04685	pectate lyase superfamily protein of *Leeuwenhoekiella palythoae*	68.11%
	DMZ48_04700	glycoside hydrolase family protein of *Galbibacter marinus*	72.09%
	DMZ48_09805	endopolygalacturonase of *Joostella marina* DSM 19592	73.56%
GH43 sub-family 4	DMZ48_01145	arabinan endo-1,5-alpha-L-arabinosidase of *Gramella* sp. SH35	70.33%
GH43 sub-family 5	DMZ48_01135	arabinan endo-1,5-alpha-L-arabinosidase of *Flavobacteriaceae* bacterium FS1-H7996/R	71.74%
GH43 sub-family 37 with CBM61	DMZ48_08955	beta-xylosidase of *Zunongwangia atlantica* 22II14-10F7	67.70%
GH53	DMZ48_07175	arabinogalactan endo-1,4-beta-galactosidase of *Flaviramulus basaltis*	68.39%
GH88	DMZ48_16440	glycosyl hydrolase family 88 of *Joostella marina* DSM 19592	71.96%
GH105	DMZ48_02835	putative unsaturated glucuronyl hydrolase of *Joostella marina* DSM 19592]	71.13%
	DMZ48_04715	family 88 glycosyl hydrolase of *Flavobacterium* sp. IMCC34759	75.40%
GH106	DMZ48_04710	glycoside hydrolase of *Galbibacter marinus*	60.57%
GH145	DMZ48_16445	neuraminidase of *Flavobacterium daejeonense*	61.00%
GH127	DMZ48_01130	hypothetical protein SAMN05216294_1503 of *Muricauda zhangzhouensis*	68.92%
	DMZ48_14480	DUF1680 family protein of *Larkinella arboricola*	60.88%
	DMZ48_15545	hypothetical protein JoomaDRAFT_0361 of *Joostella marina* DSM 19592	64.77%
GH146	DMZ48_12780	protein of unknown function DUF1680 of *Muricauda ruestringensis* DSM 13258	72.94%
CE12	DMZ48_02845	rhamnogalacturonan acetylesterase of *Flaviramulus basaltis*	64.11%
	DMZ48_04660	rhamnogalacturonan acetylesterase of *Sinomicrobium pectinilyticum*	56.08%
CE12 + CE12	DMZ48_04670	Lysophospholipase L1 of *Salegentibacter salinarum*	64.53%
CE12 + CE10	DMZ48_09795	esterase/lipase of *Joostella marina* DSM 19592	61.72%

PL1 sub-family 2	DMZ48_09815	hypothetical protein JoomaDRAFT_1172 of *Joostella marina* DSM 19592	68.08%
PL1 sub-family 2 + CE8	DMZ48_09810	Pectinesterase A precursor of *Mariniflexile* sp. TRM1-10	65.91%
PL10 + CE8	DMZ48_02840	pectinesterase of *Maribacter polysiphoniae*	53.28%
PL22	DMZ48_04705	hypothetical protein C7S20_10175 of *Gramella* sp. SH35	59.17%
CBM4	DMZ48_13415	hypothetical protein of *Lewinella nigricans* DSM 23189	46.24%
CBM9	DMZ48_08015	Carbohydrate family 9 binding domain of *Leeuwenhoekiella palythoae*	56.61%

Table S3. Putative horizontal transferred genes of *R. solimangrovi* related to lignocellulose degradation, inferred from genomic data.

Enzyme family	Gene annotation	Origin
GH2	β-galactosidase	*Tangfeifania diversioriginum* DSM 27063
GH5 sub-family 4	Xyloglucan-specific endo-β-1,4-glucanase	*Prolixibacter denitrificans* DSM 27267
GH9	Endoglucanase/ exoglucanase	*Draconibacterium orientale* DSM 25947
GH16	Xyloglucan-specific endo-β-1,4-glucanase	*Chitinophagaceae* bacterium PMP191F
GH43	β-xylosidase	*Echinicola vietnamensis* KMM 6221
GH43 sub-family 17	α-L-arabinofuranosidase	*Cyclobacterium lianum* CGMCC 1.6102
GH43 sub-family 28	Uncharacterized xylanase	*Porphyromonadaceae* bacterium KH3CP3RA
GH127	β-L-arabinofuranosidase	*Larkinella arboricola* DSM 21851

Table S4. List of potential genes involved in uptake and metabolism of sugars, inferred from genome of *R. solimangrovi*.

Category	Gene annotation	Number of genes	NCBI accession
Sugar transporters	Na^+/glucose cotransporter	3	TRZ45902<br>TRZ42504<br>TRZ42552
	ABC transporter	9	TRZ46117<br>TRZ45189<br>TRZ45190<br>TRZ44474<br>TRZ43527<br>TRZ43218<br>TRZ42752<br>TRZ42238<br>TRZ42030
	MFS transporter	16	TRZ45892<br>TRZ46281<br>TRZ45171<br>TRZ43987<br>TRZ43544<br>TRZ43734<br>TRZ43151<br>TRZ43247<br>TRZ43248<br>TRZ43275<br>TRZ43297<br>TRZ42558<br>TRZ42286<br>TRZ41998<br>TRZ41888<br>TRZ41890
	MFS transporter AraJ	1	TRZ41988
	Glucose/galactose MFS transporter	1	TRZ42054
	EamA/RhaT family transporter	5	TRZ45859<br>TRZ43560<br>TRZ42617<br>TRZ41572<br>TRZ41062
Glucose conversion	Polyphosphate glucokinase	1	TRZ42724
	Glucose-6-phosphate isomerase	1	TRZ45841
Mannose conversion	Carbohydrate kinase	1	TRZ45050
	Mannose-6-phosphate isomerase	1	TRZ41776
Galactose conversion	Galactose-1-epimerase	4	TRZ42808<br>TRZ42233<br>TRZ42045<br>TRZ45903

	Galactokinase	2	TRZ44284<br>TRZ44304
	Galactose-1-phosphate uridylyltransferase	1	TRZ44283
	phospho-sugar mutase	1	TRZ43683
Xylose conversion	Xylose isomerase	1	TRZ42503
	Xylulokinase	1	TRZ42502
Arabinofuranose conversion	Arabinose isomerase	1	TRZ45904
	Ribulokinase	1	TRZ45906
	Ribulose-5-phosphate 4-epimerase	1	TRZ45905
Pentose phosphate pathway	Transketolase	2	TRZ41575<br>TRZ41576
